# Community structure of soil fungi in a novel perennial crop monoculture, annual agriculture, and native prairie reconstruction

**DOI:** 10.1371/journal.pone.0228202

**Published:** 2020-01-30

**Authors:** Thomas P. McKenna, Timothy E. Crews, Laura Kemp, Benjamin A. Sikes

**Affiliations:** 1 University of Kansas, Lawrence, Kansas, United States of America; 2 The Land Institute, Salina, Kansas, United States of America; Feroze Gandhi Degree College, INDIA

## Abstract

The use of perennial crop species in agricultural systems may increase ecosystem services and sustainability. Because soil microbial communities play a major role in many processes on which ecosystem services and sustainability depend, characterization of soil community structure in novel perennial crop systems is necessary to understand potential shifts in function and crop responses. Here, we characterized soil fungal community composition at two depths (0–10 and 10–30 cm) in replicated, long-term plots containing one of three different cropping systems: a tilled three-crop rotation of annual crops, a novel perennial crop monoculture (*Intermediate wheatgrass*, which produces the grain Kernza^®^), and a native prairie reconstruction. The overall fungal community was similar under the perennial monoculture and native vegetation, but both were distinct from those in annual agriculture. The mutualist and saprotrophic community subsets mirrored differences of the overall community, but pathogens were similar among cropping systems. Depth structured overall communities as well as each functional group subset. These results reinforce studies showing strong effects of tillage and sampling depth on soil community structure and suggest plant species diversity may play a weaker role. Similarities in the overall and functional fungal communities between the perennial monoculture and native vegetation suggest Kernza^®^ cropping systems have the potential to mimic reconstructed natural systems.

## Introduction

Intensive agricultural practices can have many detrimental environmental impacts and reduce important ecosystem services [1,2]. To offset these effects and increase the sustainability of agricultural systems, there has been a push to better mimic natural systems, such as intact prairies and grasslands, by growing perennial crop species [3–5]. Long-lived crop species have the potential to improve water and soil conservation, improve overall soil health, and reduce inputs compared to conventional agriculture practices [6]. Perennial crops can alter abiotic soil properties [7,8], but much less is known about how perennial crop species impact soil microbial community structure. Soil communities underlie many of the ecosystem processes on which sustainability relies [9,10], so understanding community differences among agricultural and natural systems is critical to increase sustainability.

Agricultural systems implement many cropping practices that act as abiotic and biotic filters of microbial communities [11–14]. Repeated soil disturbance, such as in a conventionally tilled agriculture system, often leads to changes in the diversity and overall community structure of soil communities [11,15]. Nutrient enrichment to increase or maintain productivity shifts microbial dominance from slow to fast growing taxa and may decrease abundance of plant mutualists, with important implications for nutrient cycling and retention [16]. Variation in plant diversity (temporal and spatial [17–19]) and plant longevity (annual vs perennial plants [4,20]) among cropping systems changes the abundance and diversity of plant derived resources, which can also lead to changes in soil community structure. Fungal communities contribute to the stability and productivity of natural and agricultural systems [9], so understanding cropping system impacts on fungal community structure is critical to predict their impact on plants. Mutualistic fungi form symbioses on which the vast majority of plants rely and saprotrophic fungi play a major role in decomposition and nutrient cycling [21]. Moreover, fungal pathogens maintain productivity in natural systems, balancing the relationship between plant diversity and productivity [22,23] and represent diseases that cause great losses in agroecosystems [24].

Tillage in annual cropping systems alters soil physical structure and resource availability, which can alter soil fungal community as a whole and shift fungal functional groups (pathogens, saprotrophs, and mutualists) [15,25,26]. Increased incorporation of plant residues with tillage may reduce fungal pathogens in annual cropping systems [27,28], while the physical cutting and disruption of hyphal formation can have varying effects on saprotrophs [25,29,30] and decrease abundance of mutualists [25,29–31].

The plant community in a cropping system provides resources for soil fungal communities as hosts for pathogens and mutualists and through litter and root inputs for saprotrophs. The quality and quantity of host and input resources may depend on the characteristics of the crop (identity, functional group, and life cycle) planted as well as temporal and spatial crop diversity. For instance, legume crops may be colonized by a unique community of fungal mutualists [32], have unique root architecture [33], and cause increased nutrient availability [34] compared to non-legume crops, but these functional group effects may also vary among legume species [35,36]. Resources may also change with the life cycle of a crop. Crops with a perennial life cycle typically have greater belowground productivity than annual crops, which may increase the vertical distribution and total pool of root associated resources [37,38]. Variations in crop characteristics lead to diversification of resources (litter and root inputs) and potential hosts for soil fungi when crop diversity is increased. This increase can lead to changes in the overall fungal community and fungal functional groups [17,19,36,39].

Fungal community changes in response to management shifts among cropping systems may also differ markedly with soil depth. Fungal communities have been shown to differ with sampling depth [40,41], but the depth at which communities change may depend on how a cropping system is managed. In a cropping system with tillage, homogenization of the upper soil layers may lead to similar fungal communities throughout the entirety of disturbed soil [14,25]. In cropping systems without tillage, distinct fungal communities may be found at shallower depths due to a maintained gradient of resources [25,42].

To better understand how a novel perennial cropping system affects soil community structure, we characterized soil fungal communities in the Agroecology Research Trials at the Land Institute in Salina, KS. Soil samples were taken from conventionally tilled annual plots, novel perennial crop (*Intermediate wheatgrass*) monoculture plots, and reconstructed native prairie plots. Samples were taken at two depths to assess how management practices effects on fungi may differ with depth. We expected each cropping system to have a unique fungal community due to tillage and temporal diversity in the annual system, no soil disturbance and low plant diversity in the perennial monocrop, and no soil disturbance and high plant diversity in the native prairie polyculture. We also expected the differences in plant communities and disturbance to create unique fungal functional group (pathogens, saprotrophs, mutualists) communities.

## Methods

### Site description

The Agroecology Research Trials (A.R.T. plots) were established at the Land Institute in Salina, Kansas in 2002. The experimental site soil is classified as McCook series Fluventic Haplustoll that had been in alfalfa production since 1996. The existing alfalfa stand was removed using an under-cutter and disc harrow before experiment installation.

### Experiment layout

Research blocks were separated into plots (ca. 900 m^2^ with no aisles), and one of three cropping systems was randomly assigned to each plot within each block. The cropping systems were: perennial monoculture, native vegetation, and annual agriculture. Perennial monocultures consist of intermediate wheatgrass (*Thinopyrum intermedium*), which produces the perennial grain Kernza®. Thus, each block contains a representative plot of each cropping system. Plots have been repeatedly harvested for both seed and forage. Some invasion by native warm-season grasses has occurred in recent years. The native vegetation plots were planted with a native prairie seed mix consisting of warm-season grasses, cool-season grasses, and forbs to reconstruct a native prairie system. Plots are managed as hay meadows with frequent swathing and baling and occasional burning. In recent years the native plant community has been dominated by the grasses big bluestem (*Andropogon gerardii*), Indiangrass (*Sorghastrum nutans*), switchgrass (*Panicum virgatum*), eastern gamagrass (*Tripsacum dactyloides*), sideoats grama (*Bouteloua curtipendula*), interspersed with the legume Illinois bundleflower (*Desmanthus illinoensis*), and the forb Maximillian sunflower (*Helianthus maximiliani*). Some plots have been invaded by smooth bromegrass (*Bromus inermis*).

Annual agriculture plots are on a rain-fed winter wheat—sorghum—soybean crop sequence. Nitrogen (~ 84–123 kilograms/hectare) and phosphorus (~ 56 kilograms/hectare) fertilizers are applied as needed before planting of crops. Plots are tilled annually with a disc, chisel, and or harrow before planting. A cultivator is used as needed for weed control.

### Soil samples

Soil samples (1.7 x 30 cm) were taken from each cropping system plot across the three blocks in June 2015. At the time, annual agriculture plots were planted to winter wheat. Three cores were taken from each plot at least 2m away from the plot edge, separated by depth into 0–10 cm and 10–30 cm subsamples, then subsamples from the same depth were pooled. Final soils for analysis included pooled samples from each depth, for each cropping system, replicated across the three blocks (2 depths X 3 cropping system plots X 3 blocks). Soils were stored at -20°C for 2 months until processing.

### DNA extraction and sequencing

DNA was extracted from 0.25 g of each molecular subsample using MoBio PowerSoil Kits (MoBio, Carlsbad, USA). Extracted DNA was quantified using a Qubit 2.0 (LifeTechnologies, Carlsbad, USA) to normalize template DNA for PCR. The ITS2 region of rDNA was amplified from 5 ng of template DNA with fungal-specific primers ITS4 and fITS7 using the PCR parameters described in Ihrmark et al. (2012) [43] and Q5 proof-reading polymerase (New England Biosystems, Ipswich, USA). PCR products were cleaned using Agencourt AMPure XP magnetic beads (Beckman Coulter, Indianapolis, USA), then unique Nexterra indices (Illumina, San Diego, USA) were ligated to each sample. Finally, a second bead-purification step was used. Final libraries for each sample were pooled to a single library; concentration and size were verified using a TapeStation 2200 (Agilent, Santa Clara, USA). Fungal sequences were generated using an Illumina Mi-seq (Illumina, San Diego, USA) at the Kansas State Integrated Genomics Center. Qiime v.1.9.0 was used for bioinformatic processing [44]. Quality and barcode filtering resulted in 2,281,565 reads with an average phred score ≥30 and median length of 270 bp. Open-reference OTU picking and chimera checking using Usearch 6.1 and the UNITE fungal ITS reference database [45] was used to cluster OTUs (97%). All OTUs with <5 reads were removed to eliminate potential PCR/sequencing artifacts. Taxonomic classification was assigned to OTUs using the RDP classifier. The DESeq2 variance-stabilizing transformation [46] was performed in R to normalize the data [47]. Because of the debate about how to normalize microbiome data [46,48,49], the dataset was also rarefied to equal sampling depth. The statistical analysis described below was performed on the DESeq2 and rarefied data. The results were qualitatively similar, so for brevity only the DESeq2 results are presented. FUNguild [50] was used to assign functional classification to OTUs. LINUX code for the entire bioinformatics pipeline is available by request. Sequences were submitted to the National Center for Biotechnology Sequence Read Archive under #PRJNA528501. Library preparation and bioinformatics were completed in collaboration with the KU Center for Metagenomic Microbial Community Analysis and the KU Advanced Computing Facility.

### Statistical analyses

Analyses were performed on all OTUs before FUNguild (All) and OTUs solely designated as “highly probable” and “probable” Pathotrophs, Saprotrophs, and Symbiotrophs (mutualists) in FUNguild. Nonmetric multidimensional (NMS) scaling with the Sørensen index was used to visually inspect for the differences in fungal communities among cropping systems and sample depth (PC-ORD 6.0, MjM Software Design, Gleneden Beach, OR, USA). Permutational MANOVAs (PerMANOVA, 999 permutations) with the Bray-Curtis dissimilarity index were performed using the vegan package in R [51] to test for differences among treatment groups, and significant PerMANOVAs were followed by a pairwise comparison test using the RVAideMemoire package in R [52]to identify differences among treatments. The inverse Simpson’s Diversity (1/D) and observed richness of OTUs within all, pathotroph, saprotroph, and symbiotroph categories were compared among treatment groups using ANOVA (proc glm; SAS^™^ v9.3; SAS Institute Inc., Cary, NC, USA) with block, planting treatment, and depth as fixed factors. Significant ANOVAs were followed by a Tukey’s HSD multiple comparison test to identify differences among treatments. The results of the analysis of the diversity indices and observed richness were similar for all categories, so for brevity only the richness results are presented (see Supplement S1 Table, S1 Fig, and S2 Fig for diversity results). Blocked indicator species analysis was performed on all OTUs to identify representative OTUs at each depth within each cropping system (PC-ORD 6.0, MjM Software Design, Gleneden Beach, OR, USA).

## Results

The bioinformatics analysis resulted in 7496 fungal OTUs. The phylum with the greatest OTU richness was Ascomycota (79.7%) followed by unclassified OTUs (10.4%), then Basidiomycota (5.2%), Glomeromycota (2.9%), Zygomycota (1.48%), and Chytridiomycota (0.4%). Unidentified OTUs had the greatest abundance, followed by Ascomycota, Glomeromycota, Basidiomycota, Zygomycota, and Chytridmycota. The relative abundance of each phylum generally followed this pattern, but slightly varied among cropping system and sampling depth (Table 1). FUNguild assigned an ecological function to 2399 OTUs (32.0%).

**Table 1:** Relative abundance (percent) of fungal phyla OTUs in each cropping system (annual agriculture (AN), native vegetation (NV), and perennial monoculture (PM)) and at each sampling depth (0–10 cm (10) and 10–30 cm (30)).

Cropping system and depth	Unidentified	Ascomycota	Glomeromycota	Basidiomycota	Zygomycota	Chytridmycota
PM10	50.45	43.12	2.99	2.46	0.66	0.33
PM30	53.02	36.22	5.97	2.87	1.64	0.28
AN10	48.12	45.14	1.65	3.48	1.17	0.43
AN30	46.19	42.27	3.54	2.18	5.50	0.32
NV10	48.23	44.91	2.82	2.58	0.87	0.60
NV30	54.18	37.28	3.80	2.12	2.15	0.47

**Fig 1:**
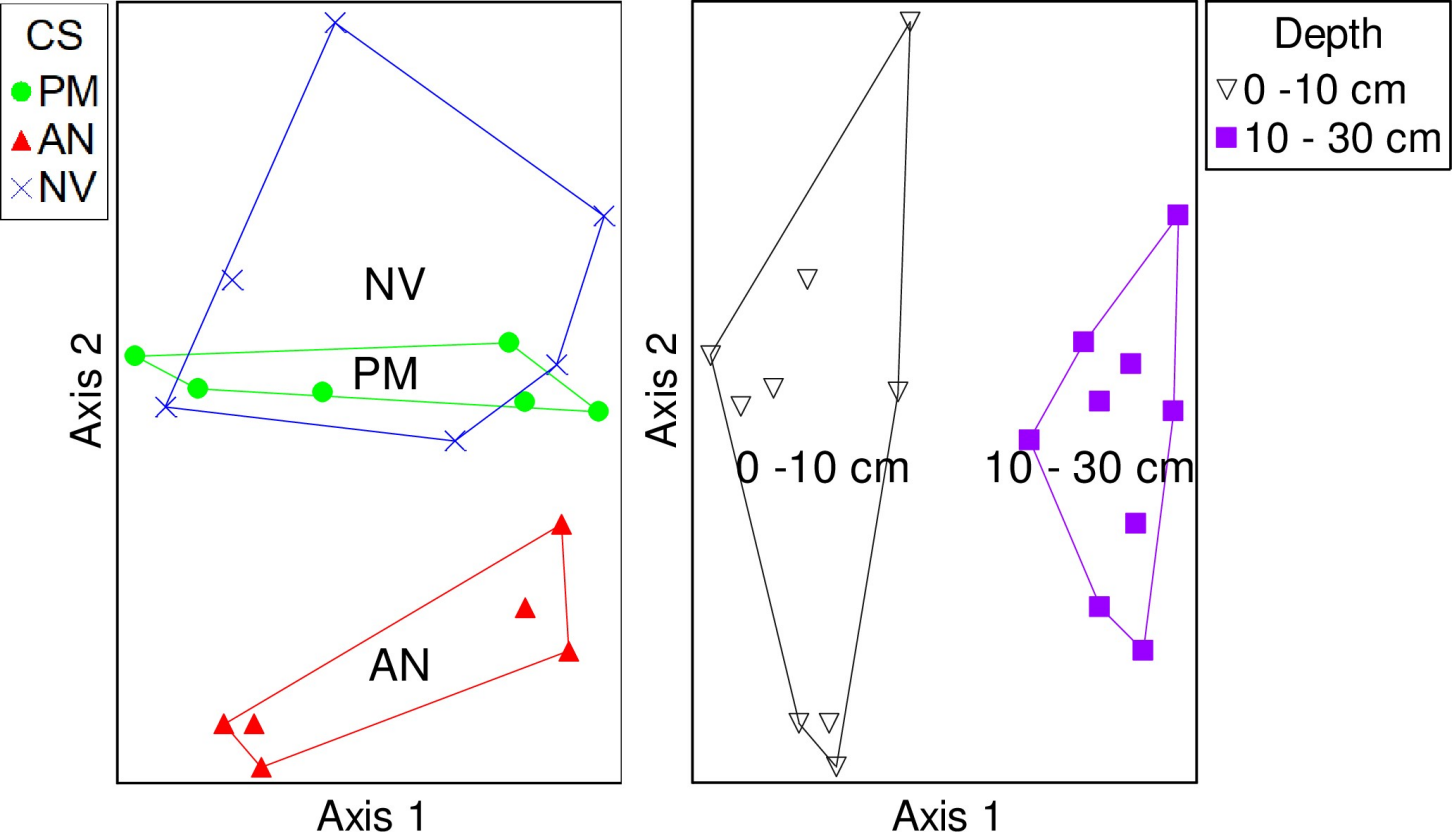
Nonmetric multidimensional scaling ordination of All OTUs sampled from plots of the three cropping systems (CS: perennial monoculture (PM), annual agriculture (AN), native vegetation (NV); A) and two soil depths (0–10 and 10–30 cm; B). The cropping system or depth label is at the centroid of each community.

**Fig 2:**
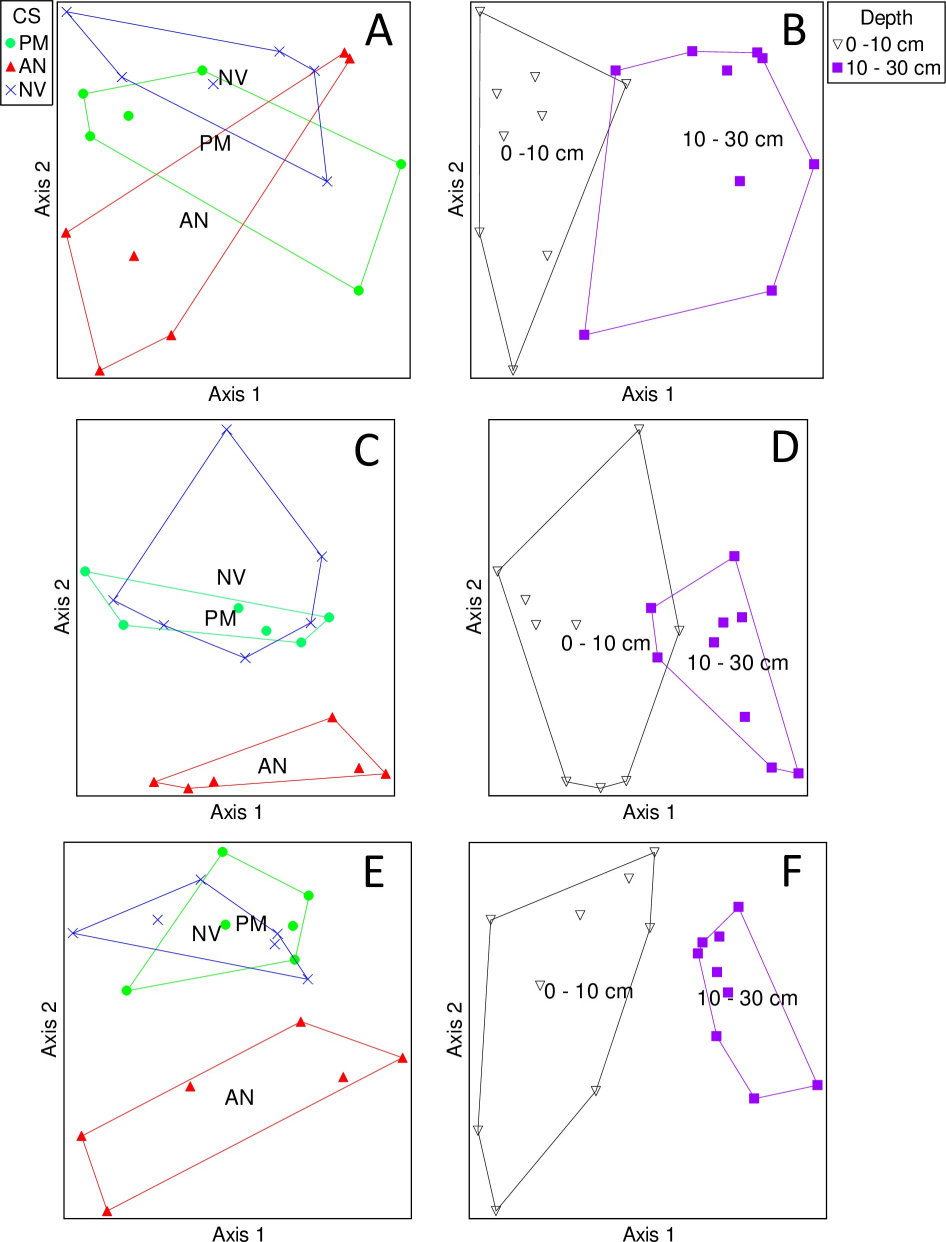
Nonmetric multidimensional scaling ordination of Pathotroph (A,B), Saprotroph (C,D), and Symbiotroph (E,F) OTUs sampled from plots of three cropping systems(CS: perennial monoculture (PM), annual agriculture (AN), native vegetation (NV)) and two soil depths (0–10 and 10–30 cm). The cropping system or depth label is at the centroid of each community.

Community analysis using all OTUs revealed that the soil fungal communities were similar in cropping systems with perennial monocultures of intermediate wheatgrass and native vegetation, and both were different than the annual agriculture system (Table 2, Fig 1A). Soil fungal communities also differed at 0–10 and 10–30 cm within each cropping system (Table 2, Fig 1B). Analysis of OTUs assigned to a functional classification showed that pathotroph communities were similar among cropping systems but differed at the two sampling depths (Table 2; Fig 2A and 2B). Saprotroph and symbiotroph communities were similar in the intermediate wheatgrass and native vegetation cropping systems, and both were different than annual agriculture system (Fig 2C–2E). Saprotroph and symbiotroph communities also differed with sampling depth (Fig 2D–2F).

**Table 2:** Degrees of freedom (DF), pseudo F values, and R^2^ from PerMANOVAs for the effect of three cropping systems (perennial monoculture (PM), annual agriculture (AN), and native vegetation (NV)) and two soil depths (0–10 and 10–30 cm) on soil fungal communities. All consists of the entire fungal community before using FUNguild and the three trophic guilds (Pathotroph, Saprotroph, and Symbiotroph) consist of OTUs categorized as “highly probable” and “probable” by FUNguild. Pairwise comparisons were made among cropping systems across depths for each PerMANOVA (different letters indicate a significant difference (p < 0.05)).

		All		Pathotrophs		Saprotrophs		Symbiotrophs	
	DF	F	R^2^	F	R^2^	F	R^2^	F	R^2^
Block	2,10	1.67**	0.12	0.94**	0.09	1.98**	0.14	1.84**	0.13
Cropping System (CS)	2,10	3.09**	0.23	1.38	0.13	3.36**	0.24	3.48**	0.25
Depth	1,10	4.93**	0.18	4.00**	0.19	4.43**	0.16	5.68**	0.20
CS*Depth	2,10	1.50†	0.11	1.25	0.12	1.20	0.09	1.00	0.07
CS pairwise comparison	PM	a		a		a		a	
	AN	b		a		b		b	
	NV	a		a		a		a	

† p < 0.10

* p < 0.05, and

** p < 0.01

**Fig 3:**
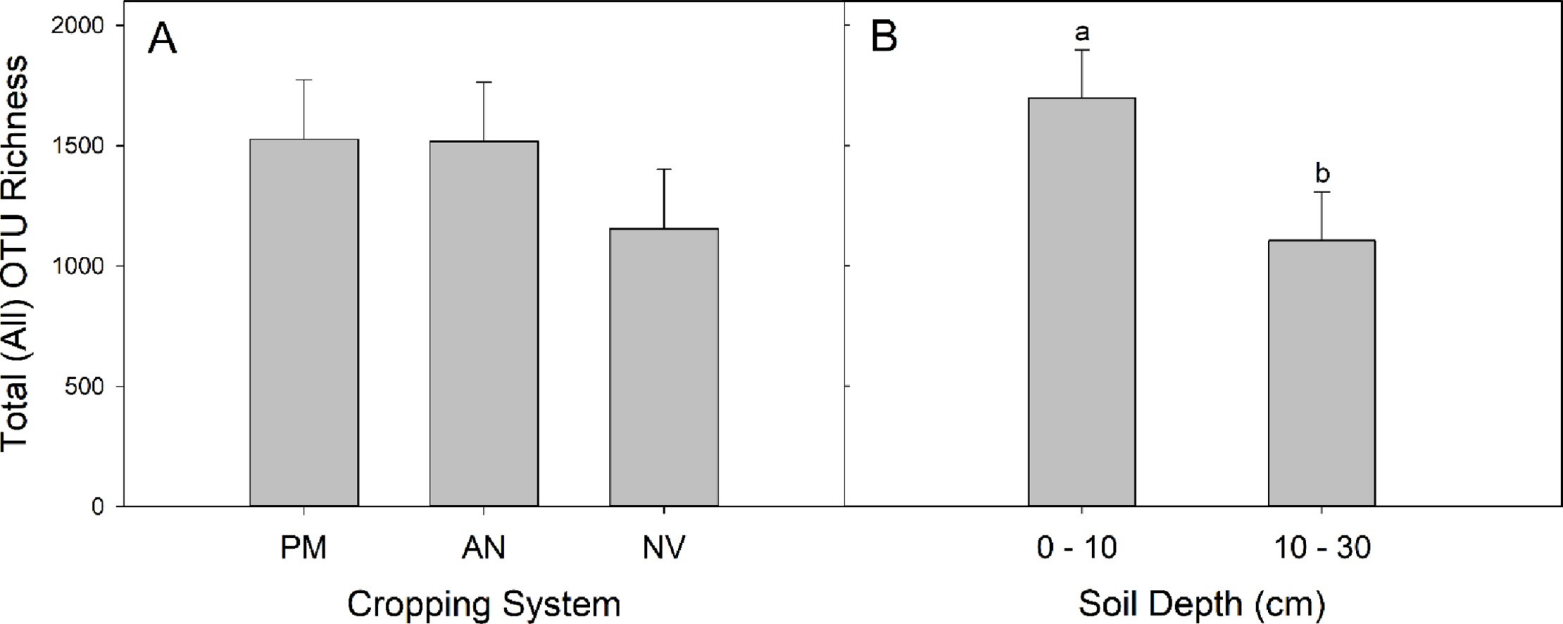
Richness (Least Square mean ± 95% confidence limits) of All OTUs across the three cropping systems (perennial monoculture (PM), annual agriculture (AN), and native vegetation (NV); A) and each sampling depth across cropping systems (B). Different letters indicate a significant difference (Tukey’s HSD multiple comparison)). The ANOVA was marginally significant for cropping system (A; Table 2), so no multiple comparisons test was performed.

**Fig 4:**
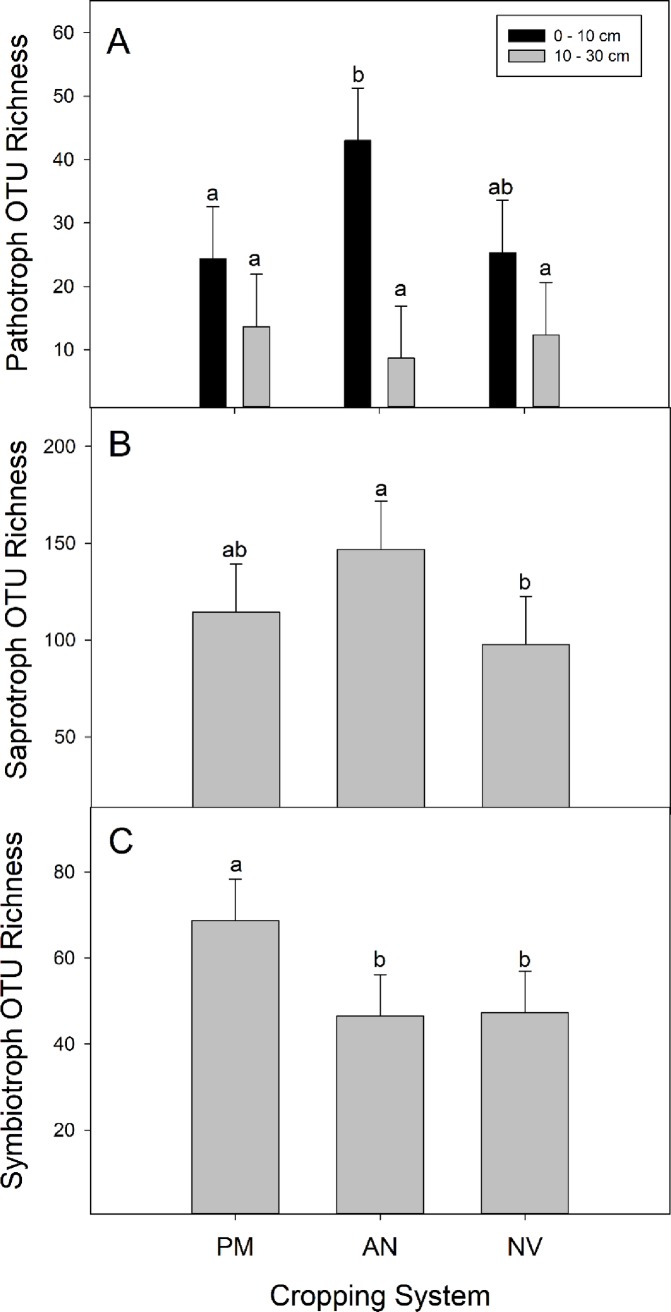
Richness (Least Square mean ± 95% confidence limits) of Pathotroph OTUs in each cropping system (perennial monoculture (PM), annual agriculture (AN), and native vegetation (NV) and at each sampling depth (0–10 cm and 10–30; significant cropping system x depth interaction; A) and Saprotroph (B) and Symbiotroph (C) OTUs from the three cropping systems across sampling depths (significant main effect of cropping system). Different letters indicate a significant difference (Tukey’s HSD multiple comparison).

The richness of all OTUs (All) was marginally greater in perennial monocultures and annual agriculture than native vegetation (main effect of cropping system; Table 3; Fig 3A), and richness decreased with depth (main effect of depth; Fig 3B). Pathotroph richness decreased with depth but only significantly in the annual agriculture system (cropping system x depth interaction; Fig 4A). Saprotroph richness was greater in annual agriculture than the native vegetation (Table 3, Fig 4B), and the richness of symbiotrophs was greatest in perennial monocultures (Fig 4C).

**Table 3:** ANOVA (degrees of freedom (DF) and F values) results for the effect of block, cropping system (perennial monoculture, annual agriculture, and native vegetation), and sample depth (0–10 and 10–30 cm) on richness of All, Pathotroph, Saprotroph, and Symbiotroph fungal OTUs.

Variable	DF	All	Pathotrophs	Saprotrophs	Symbiotrophs
Block	2,10	0.58	0.89	0.57	4.46*
Cropping System (CS)	2,10	3.68†	2.32	5.00*	8.47**
Depth	1,10	21.42**	40.84**	2.13	0.06
CS*Depth	2,10	2.26	6.20*	0.03	0.20

† p < 0.10

* p < 0.05, and

** p < 0.01

Indicator species analysis performed on all OTUs identified 612 significant indicator OTUS, and 94 had highly probable and probable classifications (S2 Table). At the 0–10 cm depth, the perennial monoculture system had 9 OTUs with highly probable and probable classifications. The majority were saprotrophs (5 OTUs) in the phylum Ascomycota, followed by symbiotrophs (3 OTUs) in the genus *Glomus* and a single pathotroph in the phylum Ascomycota and genus *Leptoshpaeria*. The annual agriculture systems had 34 highly probable and probable classifications with a majority being saprotrophs (29 OTUs), followed by pathotrophs (4 OTUs), and a single symbiotroph from the genus *Funneliformis*. The native vegetation system had one highly probable saprotroph from the phylum Ascomycota and genus *Pyrenochaeta*. At the 10–30 cm depth, perennial monocultures had 16 highly probable and probable classified OTUs. All were symbiotrophs with three identified to the genus *Glomus*. The annual agriculture system had 33 highly probable and probable classified OTUs with a majority being saprotrophs (27 OTUs) in the phylum Zygomycota (17 OTUs) followed by symbiotrophs (6 OTUs). A single symbiotroph was identified to the genus *Septoglomus*. The native vegetation system had a single highly probable saprotroph in the phylum Zygomycota and genus *Ramicandelbar*.

## Discussion

The objectives of this study were to characterize and compare the soil fungal community at two depths in long term plots conditioned by three different cropping systems. The overall soil fungal community, saprotrophs, and symbiotrophs were similar in perennial monoculture and native vegetation cropping systems, but both were different than the annual agriculture system. These fungal communities also differed between sampling depths within each cropping system. Pathotroph communities were similar among cropping systems, but differed between sampling depths. Overall richness (all) of OTUs decreased with depth and was lower in native vegetation than perennial monocultures and annual agriculture. Pathotroph richness decreased with depth in annual agriculture, saprotroph richness was greater in annual agriculture than native vegetation, and symbiotroph richness was greatest in perennial monocultures. Contrary to our expectations, the three cropping systems did not create three distinct soil communities, and soil fungal communities in native vegetation treatments were not more diverse than perennial monocultures. These results reinforce studies that show tillage [15,25] and depth [26,40,41] as drivers of soil community structure, and suggest that plant species diversity may play a weaker role.

The annual agriculture system had unique overall, saprotroph, and symbiotroph fungal communities. This was expected as repeated tillage [15,25] and nutrient addition [16] can have strong effects on the overall fungal community as well as its’ functional components. Indicator species analysis pointed to the unique effects of conventionally tilled agriculture on saprotrophs in the annual agriculture system, as 84% of the highly probable and probable indicator OTUs were saprotrophs. Tillage effects on saprotrophs can vary from positive [25,30] to negative [29,53,54] depending on community characterization methods, and in this study we found an increase in saprotroph richness in a system with added nutrients and tillage compared to the native vegetation plots. Tillage and nutrients typically negatively affect symbiotroph (AMF) communities [16,25,54–56], but here we found AMF richness was similar in the annual agriculture plots and in the native vegetation plots.

An increased number of plant species in the native vegetation cropping system did not support different overall or functional communities of soil fungi compared to the perennial monoculture system. Similar fungal communities have been observed when comparing long-term diverse plantings of prairie species and monocultures of the warm-season grass *Panicum virgatum* (switchgrass) in bioenergy studies [12]. In long-term grassland biodiversity experiments, increasing plant species richness effects on soil fungal communities have varied. Dassen et al. 2017 [19] sampled bulk soils along a large plant species richness gradient (1 to 60 species) at the Jena experiment in Germany. There was no effect of increasing plant species richness on overall fungal community structure, and plant functional group (grass, legume, or forb) had a greater influence on soil communities than plant species diversity (richness and abundance). These findings are very relevant to the present study, as the high abundance of grass species in the native vegetation cropping system may have contributed to similarities in fungal community composition with the perennial monoculture, as *T*. *intermedium* is also a grass. Differences in fungal community structure have been observed when comparing a subset of plots from a long term biodiversity experiment at Cedar Creek Minnesota U.S.A. Leblanc et al. 2015 [18] compared rhizosphere fungal community structure in monocultures of a grass and legume species and 16 species mixtures. Fungal community structure in the monocultures differed from each other and both differed from the rhizosphere communities in the 16 species mixtures. Both of the above studies also highlighted the importance of abiotic soil factors at the time of sampling, which unfortunately is lacking in our study.

Increased plant species richness in the native vegetation cropping system also did not lead greater overall fungal richness compared to the perennial monoculture cropping system. Previous studies have found no [57], marginal [19], and positive [18] effects of increasing plant species richness on measures of overall soil fungal diversity. The inconsistency of results among studies may be due to differences in sampling effort and type of soil samples taken (bulk soil, rhizosphere, or root) [19,57]. Interestingly, the perennial monocultures had a greater richness of symbiotrophs despite the native vegetation cropping system having greater number of potential plant hosts. AMF diversity has been shown to increase with plant diversity [39], but studies suggest that plant identity, functional group, and soil abiotic properties may be just as important [19,32]. Monitoring of abiotic properties in these cropping systems has shown conditions to be fairly similar over time (Crews unpublished data), so the difference may be due to the identity of the species in monoculture. This finding, as well as the majority of indicator OTUs (76%) being symbiotrophs, gives promise for increased ecosystem services provided by AMF and potentially increased sustainability of the perennial grain Kernza^®^.

Soil depth was also an important factor in structuring the community structure of soil fungi in this experiment. Resources tend to be higher at the soil surface and decline with depth within the soil profile [40]. This gradient tends to concentrate soil fungi at the surface and can lead to changes in community composition and diversity (richness and relative abundance) with depth [41].This is consistent with the trends observed in this experiment, as the overall and functional fungal community structure was distinct within each cropping system at each depth, and the overall OTU richness decreased with depth. Despite possible homogenization effects of tillage, distinct communities were found at both sampling depths in the annual agriculture system [14,42]. It is possible that a nutrient gradient was still present, or the deeper sample reached below the tillage depth. Of the functional classifications, pathotrophs was the only functional group to have a decrease in richness with depth. The identity and relative abundance of saprotrophs and symbiotrophs may change more than richness with depth, which lead to overall community shifts [26].

Here we characterized the overall and functional components of the soil fungal community in three cropping systems to better understand potential management practices influencing community structure and compare a novel perennial cropping system to a tilled agricultural system and reconstructed natural system. The separation of the overall and functional group communities of soil fungi in annual agriculture system from the perennial monoculture and native prairie reconstruction reinforce studies showing strong effects of tillage [15,25] and nutrient addition [16] on soil communities. Similarities in fungal community structure between the untilled perennial monoculture and native prairie reconstruction suggest the lack of a plant diversity effect, and also indicate that perennial cropping systems do have the potential to closely mimic reconstructed natural systems. Because cropping systems typically implement multiple management strategies (i.e. annual crop, tillage, and nutrient addition vs perennial crop, no-tillage, no nutrient addition), it is challenging to isolate and explicitly test specific mechanisms driving soil community structure. Additionally, these management strategies may also influence intra-seasonal variability of soil fungal communities in different ways. For example, different crops mature at varying times throughout the growing season, and crop effects fungal on community structure may differ between the onset of growth and peak biomass [58]. Future studies will focus on experiments designed to assess the relative effects of tillage, crop life cycle, and crop diversity on soil communities in these systems, as well as assess the function of the soil communities cultivated by these cropping systems by studying effects on subsequent crop growth.

## Supporting information

S1 Table ANOVA (degrees of freedom (DF) and F values) results for the effect of block, cropping system (perennial monoculture, annual agriculture, and native vegetation), and sample depth (0–10 and 10–30 cm) on the Inverse Simpson’s Diversity Index (I/D) of All, Pathotroph, Saprotroph, and Symbiotroph fungal OTUs. (DOCX)

S2 Table Results from indicator species analysis for soil samples taken from each cropping system (PM, AN, NV) and at each depth (0–10 and 10–30 cm). (DOCX)

S1 Fig Inverse Simpson’s Diversity Index (Least Square mean ± 95% confidence limits) of All OTUs from the three cropping systems (perennial monoculture (PM), annual agriculture (AN), and native vegetation (NV); A) and each sampling depth (B). Different letters indicate a significant difference (Tukey’s HSD multiple comparison)). The ANOVA was marginally significant for cropping system (A; S1 Table), so no multiple comparisons test was performed.
(DOCX)

S2 Fig Inverse Simpson’s Diversity Index (Least Square mean ± 95% confidence limits) of Pathotroph (A), Saprotroph (B), and Symbiotroph (C) OTUs from the three cropping systems (perennial monoculture (PM), annual agriculture (AN), and native vegetation (NV). Different letters indicate a significant difference (Tukey’s HSD multiple comparison).
(DOCX)
