# Supplementary material for: Community structure of soil fungi in a novel perennial crop monoculture, annual agriculture, and native prairie reconstruction
Source: PLoS One. 2020 Jan 30;15(1):e0228202. doi: 10.1371/journal.pone.0228202 (PMC6991957; doi:10.1371/journal.pone.0228202)
Supplement: S1 Table — (DOCX) [file pone.0228202.s001.docx]

**Table S1.** ANOVA (degrees of freedom (DF) and F values) results for the effect of block, cropping system (perennial monoculture, annual agriculture, and native vegetation), and sample depth (0 -10 and 10 - 30 cm) on the Inverse Simpson’s Diversity Index (I/D) of All, Pathotroph, Saprotroph, and Symbiotroph fungal OTUs.

| Variable | DF |  | All |  | Pathotrophs |  | Saprotrophs |  | Symbiotrophs |
| --- | --- | --- | --- | --- | --- | --- | --- | --- | --- |
| Block | 2,10 |  | 0.55 |  | 1.32 |  | 0.47 |  | 4.43* |
| Cropping System (CS) | 2,10 |  | 3.38† |  | 2.33 |  | 4.87* |  | 7.12* |
| Depth | 1,10 |  | 20.38** |  | 36.11** |  | 0.96 |  | 0.02 |
| CS*Depth | 2,10 |  | 2.04 |  | 5.83** |  | 0.03 |  | 0.44 |
