# Supplementary material for: Community structure of soil fungi in a novel perennial crop monoculture, annual agriculture, and native prairie reconstruction
Source: PLoS One. 2020 Jan 30;15(1):e0228202. doi: 10.1371/journal.pone.0228202 (PMC6991957; doi:10.1371/journal.pone.0228202)
Supplement: S2 Table — (DOCX) [file pone.0228202.s002.docx]

**Table S2.** Results from indicator species analysis for soil samples taken from each cropping system (PM, AN, NV) and at each depth (0 – 10 and 10 – 30 cm).

| Depth (cm) | Maxgrp | Value (IV) | Mean | S.Dev | p * | Taxon | Trophic Mode | Guild |
| --- | --- | --- | --- | --- | --- | --- | --- | --- |
| 0 - 10 | PM | 41.3 | 28.3 | 7.63 | 0.0068 | Preussia | Saprotroph | Undefined Saprotroph |
|  | PM | 72.6 | 31.8 | 15.03 | 0.0152 | Paraconiothyrium | Saprotroph | Undefined Saprotroph |
|  | PM | 89.6 | 30 | 17.72 | 0.0292 | Glomeraceae | Symbiotroph | Arbuscular Mycorrhizal |
|  | PM | 85.6 | 31.4 | 16.67 | 0.0292 | Glomeraceae | Symbiotroph | Arbuscular Mycorrhizal |
|  | PM | 78.7 | 32.3 | 15.2 | 0.0292 | Preussia | Saprotroph | Undefined Saprotroph |
|  | PM | 68.9 | 32.1 | 14.79 | 0.0292 | Glomeraceae | Symbiotroph | Arbuscular Mycorrhizal |
|  | PM | 76.4 | 33.7 | 14.91 | 0.0292 | Preussia | Saprotroph | Undefined Saprotroph |
|  | PM | 44.6 | 32.2 | 7.45 | 0.0324 | Leptosphaeria | Pathotroph | Plant Pathogen |
|  | PM | 71.9 | 31 | 14.32 | 0.049 | Preussia | Saprotroph | Undefined Saprotroph |
|  | AN | 69.9 | 34.6 | 14.5 | 0.022 | Ascobolaceae | Saprotroph | Dung Saprotroph-Soil Saprotroph-Wood Saprotroph |
|  | AN | 47.4 | 31 | 11.34 | 0.0252 | Phaeosphaeria | Saprotroph | Undefined Saprotroph |
|  | AN | 31.2 | 26.2 | 3.21 | 0.028 | Preussia | Saprotroph | Undefined Saprotroph |
|  | AN | 35.1 | 27.5 | 6.97 | 0.0288 | Chrysosporium | Saprotroph | Undefined Saprotroph-Wood Saprotroph |
|  | AN | 71.1 | 31.8 | 13.88 | 0.0296 | Humicola | Saprotroph | Undefined Saprotroph-Wood Saprotroph |
|  | AN | 36.5 | 28.1 | 5.13 | 0.0296 | Humicola | Saprotroph | Undefined Saprotroph-Wood Saprotroph |
|  | AN | 59.9 | 30.6 | 11.92 | 0.0296 | Myrothecium | Saprotroph | Undefined Saprotroph |
|  | AN | 100 | 27.4 | 20.64 | 0.0302 | Preussia | Saprotroph | Undefined Saprotroph |
|  | AN | 100 | 27.4 | 20.64 | 0.0302 | Volutella | Pathotroph | Plant Pathogen |
|  | AN | 100 | 27.4 | 20.64 | 0.0302 | Monographella | Pathotroph | Plant Pathogen |
|  | AN | 100 | 27.4 | 20.64 | 0.0302 | Phaeosphaeria | Saprotroph | Undefined Saprotroph |
|  | AN | 92.1 | 30.3 | 18.47 | 0.0302 | Ascobolaceae | Saprotroph | Dung Saprotroph-Soil Saprotroph-Wood Saprotroph |
|  | AN | 100 | 27.4 | 20.64 | 0.0302 | Sporormia | Saprotroph | Dung Saprotroph |
|  | AN | 100 | 27.4 | 20.64 | 0.0302 | Sporormia | Saprotroph | Dung Saprotroph |
|  | AN | 100 | 27.4 | 20.64 | 0.0302 | Sporormia | Saprotroph | Dung Saprotroph |
|  | AN | 100 | 27.4 | 20.64 | 0.0302 | Sporormia | Saprotroph | Dung Saprotroph |
|  | AN | 89.4 | 32.3 | 17.08 | 0.0302 | Sporormia | Saprotroph | Dung Saprotroph |
|  | AN | 72.9 | 30.9 | 15.31 | 0.0302 | Preussia | Saprotroph | Undefined Saprotroph |
|  | AN | 100 | 27.4 | 20.64 | 0.0302 | Ascobolaceae | Saprotroph | Dung Saprotroph-Soil Saprotroph-Wood Saprotroph |
|  | AN | 100 | 27.4 | 20.64 | 0.0302 | Sporormia | Saprotroph | Dung Saprotroph |
|  | AN | 81.6 | 32.2 | 16.52 | 0.0302 | Funneliformis | Symbiotroph | Arbuscular Mycorrhizal |
|  | AN | 100 | 27.4 | 20.64 | 0.0302 | Sporormia | Saprotroph | Dung Saprotroph |
|  | AN | 100 | 27.4 | 20.64 | 0.0302 | Sporormia | Saprotroph | Dung Saprotroph |
|  | AN | 72.3 | 34.7 | 14.62 | 0.0302 | Chaetomium | Saprotroph | Dung Saprotroph-Undefined Saprotroph-Wood Saprotroph |
|  | AN | 78.2 | 31.1 | 15.87 | 0.0302 | Pyrenophora | Pathotroph | Plant Pathogen |
|  | AN | 88.6 | 29.9 | 18.04 | 0.0302 | Coprinopsis | Saprotroph | Undefined Saprotroph |
|  | AN | 66.3 | 33.2 | 14.53 | 0.0302 | Neosetophoma | Saprotroph | Undefined Saprotroph |
|  | AN | 72.5 | 33.5 | 15.17 | 0.0302 | Myrothecium | Saprotroph | Undefined Saprotroph |
| Depth (cm) | Maxgrp | Value (IV) | Mean | S.Dev | p * | Taxon | Trophic Mode | Guild |
| 0 - 10 | AN | 75.0 | 31 | 15.52 | 0.0302 | Pyrenophora | Pathotroph | Plant Pathogen |
|  | AN | 100 | 27.4 | 20.64 | 0.0302 | Sporormia | Saprotroph | Dung Saprotroph |
|  | AN | 56.0 | 30.5 | 11.7 | 0.0302 | Zopfiella | Saprotroph | Dung Saprotroph |
|  | AN | 67.3 | 31.7 | 14.61 | 0.0364 | Phaeosphaeria | Saprotroph | Undefined Saprotroph |
|  | AN | 31.4 | 26.3 | 3.48 | 0.0438 | Preussia | Saprotroph | Undefined Saprotroph |
|  | NV | 46.3 | 32.6 | 9.17 | 0.025 | Pyrenochaeta | Saprotroph | Undefined Saprotroph-Wood Saprotroph |
| 10 - 30 | PM | 56.6 | 32.8 | 11.19 | 0.016 | Glomeraceae | Symbiotroph | Arbuscular Mycorrhizal |
|  | PM | 28.3 | 23.6 | 2.11 | 0.0182 | Glomeraceae | Symbiotroph | Arbuscular Mycorrhizal |
|  | PM | 63.0 | 32.8 | 14.55 | 0.0216 | Glomeraceae | Symbiotroph | Arbuscular Mycorrhizal |
|  | PM | 33.5 | 26.4 | 3.52 | 0.0228 | Glomeraceae | Symbiotroph | Arbuscular Mycorrhizal |
|  | PM | 43.0 | 29.3 | 7.8 | 0.0238 | Glomeraceae | Symbiotroph | Arbuscular Mycorrhizal |
|  | PM | 29.2 | 24.3 | 2.35 | 0.025 | Glomeraceae | Symbiotroph | Arbuscular Mycorrhizal |
|  | PM | 35.4 | 27.9 | 5.17 | 0.0258 | Glomeraceae | Symbiotroph | Arbuscular Mycorrhizal |
|  | PM | 40.6 | 28.5 | 8.23 | 0.0266 | Glomeraceae | Symbiotroph | Arbuscular Mycorrhizal |
|  | PM | 31.2 | 26.1 | 2.53 | 0.0266 | Glomeraceae | Symbiotroph | Arbuscular Mycorrhizal |
|  | PM | 34.0 | 27 | 4.08 | 0.0266 | Glomeraceae | Symbiotroph | Arbuscular Mycorrhizal |
|  | PM | 73.6 | 31.9 | 15.17 | 0.0268 | Glomeraceae | Symbiotroph | Arbuscular Mycorrhizal |
|  | PM | 66.1 | 31.8 | 14.69 | 0.0268 | Glomeraceae | Symbiotroph | Arbuscular Mycorrhizal |
|  | PM | 40.2 | 30 | 6.84 | 0.0268 | Glomeraceae | Symbiotroph | Arbuscular Mycorrhizal |
|  | PM | 41.5 | 30.5 | 7.14 | 0.0268 | Glomeraceae | Symbiotroph | Arbuscular Mycorrhizal |
|  | PM | 34.6 | 28 | 4.84 | 0.0268 | Glomeraceae | Symbiotroph | Arbuscular Mycorrhizal |
|  | PM | 42.5 | 30.5 | 7.26 | 0.0268 | Glomeraceae | Symbiotroph | Arbuscular Mycorrhizal |
|  | AN | 68.5 | 31.8 | 15.03 | 0.0212 | Mortierella | Saprotroph | Undefined Saprotroph |
|  | AN | 49.2 | 29.9 | 7.35 | 0.0288 | Mortierella | Saprotroph | Undefined Saprotroph |
|  | AN | 53.4 | 40 | 10.43 | 0.0288 | Scytalidium | Saprotroph | Wood Saprotroph |
|  | AN | 47.8 | 29.7 | 8.19 | 0.029 | Mortierella | Saprotroph | Undefined Saprotroph |
|  | AN | 58.0 | 35.9 | 13.53 | 0.0292 | Triangularia | Saprotroph | Undefined Saprotroph |
|  | AN | 31.6 | 24.7 | 2.83 | 0.0292 | Mortierella | Saprotroph | Undefined Saprotroph |
|  | AN | 22.9 | 20.5 | 1.22 | 0.0294 | Coniocessia | Saprotroph | Undefined Saprotroph |
|  | AN | 100 | 27.2 | 20.56 | 0.0296 | Mortierella | Saprotroph | Undefined Saprotroph |
|  | AN | 84.3 | 29.6 | 17.39 | 0.0296 | Glomeraceae | Symbiotroph | Arbuscular Mycorrhizal |
|  | AN | 100 | 27.2 | 20.56 | 0.0296 | Coniocessia | Saprotroph | Undefined Saprotroph |
|  | AN | 100 | 27.2 | 20.56 | 0.0296 | Mortierella | Saprotroph | Undefined Saprotroph |
|  | AN | 95.3 | 30.1 | 18.83 | 0.0296 | Mortierella | Saprotroph | Undefined Saprotroph |
|  | AN | 76.4 | 30.7 | 15.66 | 0.0296 | Mortierella | Saprotroph | Undefined Saprotroph |
|  | AN | 93.6 | 30.1 | 18.57 | 0.0296 | Coniocessia | Saprotroph | Undefined Saprotroph |
|  | AN | 95.4 | 30.2 | 18.87 | 0.0296 | Mortierella | Saprotroph | Undefined Saprotroph |
|  | AN | 81.9 | 31 | 16.64 | 0.0296 | Mortierella | Saprotroph | Undefined Saprotroph |
|  | AN | 75.8 | 30.8 | 14.43 | 0.0296 | Coniocessia | Saprotroph | Undefined Saprotroph |
|  | AN | 73.8 | 30.8 | 15.35 | 0.0296 | Glomeraceae | Symbiotroph | Arbuscular Mycorrhizal |
|  | AN | 84.1 | 29.5 | 17.4 | 0.0296 | Neopetromyces | Saprotroph | Undefined Saprotroph |
|  | AN | 90.6 | 29.8 | 17.97 | 0.0296 | Mortierella | Saprotroph | Undefined Saprotroph |
|  | AN | 84.7 | 30 | 17.77 | 0.0296 | Septoglomus | Symbiotroph | Arbuscular Mycorrhizal |
| Depth (cm) | Maxgrp | Value (IV) | Mean | S.Dev | p * | Taxon | Trophic Mode | Guild |
| 10 – 30 | AN | 89.6 | 32.5 | 17.25 | 0.0296 | Glomeraceae | Symbiotroph | Arbuscular Mycorrhizal |
|  | AN | 79.3 | 30.9 | 15.98 | 0.0296 | Glomeraceae | Symbiotroph | Arbuscular Mycorrhizal |
|  | AN | 76.8 | 30.6 | 15.72 | 0.0296 | Mortierella | Saprotroph | Undefined Saprotroph |
|  | AN | 63.0 | 36.1 | 13.53 | 0.0296 | Tetracladium | Saprotroph | Undefined Saprotroph |
|  | AN | 65.6 | 34.1 | 14.53 | 0.0296 | Triangularia | Saprotroph | Undefined Saprotroph |
|  | AN | 84.6 | 34 | 16.25 | 0.0296 | Mortierella | Saprotroph | Undefined Saprotroph |
|  | AN | 59.7 | 32 | 12.04 | 0.0296 | Mortierella | Saprotroph | Undefined Saprotroph |
|  | AN | 23.7 | 20.6 | 1.31 | 0.0296 | Mortierella | Saprotroph | Undefined Saprotroph |
|  | AN | 24.2 | 20.5 | 1.45 | 0.0296 | Mortierella | Saprotroph | Undefined Saprotroph |
|  | AN | 31.1 | 25.8 | 3.27 | 0.0362 | Mortierella | Saprotroph | Undefined Saprotroph |
|  | AN | 28.4 | 22.7 | 4.51 | 0.0388 | Glomeraceae | Symbiotroph | Arbuscular Mycorrhizal |
|  | AN | 34.3 | 26.4 | 3.83 | 0.0388 | Coniocessia | Saprotroph | Undefined Saprotroph |
|  | NV | 51.5 | 44.4 | 3 | 0.0286 | Ramicandelaber | Saprotroph | Undefined Saprotroph |
