# Supplementary material for: Community structure of soil fungi in a novel perennial crop monoculture, annual agriculture, and native prairie reconstruction
Source: PLoS One. 2020 Jan 30;15(1):e0228202. doi: 10.1371/journal.pone.0228202 (PMC6991957; doi:10.1371/journal.pone.0228202)
Supplement: S1 Fig — Inverse Simpson’s Diversity Index (Least Square mean ± 95% confidence limits) of All OTUs from the three cropping systems (perennial monoculture (PM), annual agriculture (AN), and native vegetation (NV); A) and each sampling depth (B). Different letters indicate a significant difference (Tukey’s HSD multiple comparison)). The ANOVA was marginally significant for cropping system (A; S1 Table), so no multiple comparisons test was performed. (DOCX) [file pone.0228202.s003.docx]

**Figure S1**. Inverse Simpson’s Diversity Index (Least Square mean ± 95% confidence limits) of All OTUs from the three cropping systems (perennial monoculture (PM), annual agriculture (AN), and native vegetation (NV); A) and each sampling depth (B). Different letters indicate a significant difference (Tukey’s HSD multiple comparison)). The ANOVA was marginally significant for cropping system (A; Table 2), so no multiple comparisons test was performed.
