# Supplementary material for: Community structure of soil fungi in a novel perennial crop monoculture, annual agriculture, and native prairie reconstruction
Source: PLoS One. 2020 Jan 30;15(1):e0228202. doi: 10.1371/journal.pone.0228202 (PMC6991957; doi:10.1371/journal.pone.0228202)
Supplement: S2 Fig — Inverse Simpson’s Diversity Index (Least Square mean ± 95% confidence limits) of Pathotroph (A), Saprotroph (B), and Symbiotroph (C) OTUs from the three cropping systems (perennial monoculture (PM), annual agriculture (AN), and native vegetation (NV). Different letters indicate a significant difference (Tukey’s HSD multiple comparison). (DOCX) [file pone.0228202.s004.docx]

**Figure S2**. Inverse Simpson’s Diversity Index (Least Square mean ± 95% confidence limits) of Pathotroph (A), Saprotroph (B), and Symbiotroph (C) OTUs from the three cropping systems (perennial monoculture (PM), annual agriculture (AN), and native vegetation (NV). Different letters indicate a significant difference (Tukey’s HSD multiple comparison).
